# T follicular helper cells and antibody response to Hepatitis B virus vaccine in HIV-1 infected children receiving ART

**DOI:** 10.1038/s41598-017-09165-6

**Published:** 2017-08-21

**Authors:** Yonas Bekele, Desalegn Yibeltal, Kidist Bobosha, Temesgen E. Andargie, Mahlet Lemma, Meseret Gebre, Eyasu Mekonnen, Abiy Habtewold, Anna Nilsson, Abraham Aseffa, Rawleigh Howe, Francesca Chiodi

**Affiliations:** 1 Department of Microbiology, Tumor and Cell Biology, Karolinska Institutet, Stockholm, Sweden; 2 Armauer Hansen Research Institute (AHRI), P.O. Box 1005, Addis Ababa, Ethiopia; 3 Department of Pharmacology, School of Medicine, Addis Ababa University, Addis Ababa, Ethiopia; 4 All Africa Leprosy, Tuberculosis and Rehabilitation Training (ALERT) center, Addis Ababa, Ethiopia; 5 Department of Woman and Child Health, Karolinska Institutet, Stockholm, Sweden

## Abstract

HBV vaccine has 95% efficacy in children to prevent HBV infection and related cancer. We conducted a prospective study in HIV-1 infected children receiving ART (n = 49) and controls (n = 63) to assess humoral and cellular responses to HBV vaccine provided with three doses under an accelerated schedule of 4 weeks apart. At 1 month post-vaccination all children, except 4 HIV-1 infected, displayed protective antibody (ab) titers to HBV vaccine; ab titers were lower in infected children (P < 0.0001). Ab titers decreased (P < 0.0001) in both HIV-1 infected and control children at 6 months. The frequency of circulating Tfh (cTFh) cells was 20.3% for controls and 20.8% for infected children prior to vaccination and remained comparable post-vaccination. Cytokine expression by cTfh cells upon activation with HBV antigen was comparable in the two groups at baseline and 1 month post-vaccination. Higher plasma levels (P < 0.0001) of CXCL13 were found in infected children which correlated with cTfh cell frequency at baseline. In conclusion, a lower ab response to HBV vaccine was measured in HIV-1 infected children. The frequency and activation profile of cTfh cells was comparable in infected children and controls suggesting that cells other than Tfh cells are responsible for impaired ab response to HBV vaccine.

## Introduction

Hepatitis B Virus (HBV) causes a life-threatening infection which can lead to hepatocellular liver carcinoma (HCC), the second leading cause of death among all cancers, or cirrhosis. HBV is highly endemic in sub-Saharan Africa and East Asia with 5–10% prevalence of chronic HBV infections. The complications of HBV infection typically affect adults; much of the burden of chronic HBV is, however, due to childhood infection. Vaccination against HBV virus has shown to effectively prevent HBV infection, perinatal HBV transmissions and up to 90% of HBV related deaths^1^; the safety and immunogenicity of vaccines differ with age, genetic background, co-morbidities, gender and type of administered vaccine. While the HBV vaccine was shown to be protective in HIV-1 seronegative individuals, HIV-1 infected individuals showed a less optimal and durable serological response to this vaccine^2^.

Administration of injectable vaccines, including HBV, results in presentation of vaccine antigens by skin dendritic cells (DCs) which initiates cascades of cellular and humoral immune responses in a special microstructure of the lymph node called germinal center (GC)^3^. In the GC, CD4+ T cells will be activated by DCs and polarize towards a T follicular helper (Tfh) cell lineage through the up-regulated expression of Bcl-6, CXCR5, ICOS and PD-1^4^; cells committed to the Tfh cell lineage also down-regulate CCR7 expression to migrate into the B cells follicle in response to CXCL13 chemo-attraction. The efficiency of T-B cells interaction within the GC is crucial for development of memory B cells and ab producing plasma cells; a potent ab response induced by HBV vaccination through B and T cell interaction will protect individuals for decades^5, 6^.

Tfh cells have been described through different lineage and differentiation markers as: CXCR5^+^CD4^+^ T cells^7, 8^, PD-1^+^CXCR5^+^ or ICOS^+^CXCR5^+^ CD4^+^ T cells^9^, CD4^+^CD45RO^+^CXCR5^+^ T cells^10^, ICOS^+^PD-1^+^CXCR3^+^ among memory CD4+ T cells^11^, CCR7^high^CXCR5^high^CCR6^high^PD-1^high^ among memory CD4+ T cells^12^ and CD4 + CD45RA-CXCR5+ in combination with CCR6 and CXCR3 to characterize Th1, Th2 and Th17 like Tfh cells^13^. Memory Tfh cells found in blood are representative of the Tfh cells found in lymphoid tissue^14, 15^; thus studying cTfh cells offers a valid approach to dissect the immunology of tissue Tfh cells, especially when examining clinical specimens.

Vaccination studies conducted in humans and in animal models showed that vaccine responses correlated with the frequency of cTfh cells. Specific ab responses induced upon influenza vaccination correlated with the frequency of ICOS + CXCR3+ T fh cells^11^ and an increase in the number of Tfh cells expressing ICOS + PD-1+ correlated with the avidity of abs to influenza vaccine^16^. Elderly people have a reduced ab response to vaccines due to a declined frequency of cTfh cells and T cell specimens from elderly people provide poor B cell help in culture^17^. Tfh cells produce cytokines, including IL-21 and IL-4, important for differentiation and maturation of B cells. Spensieri *et al*.^18^ examined the cytokine expression of cTfh cells and found that the frequency of CD4 + IL-21 + ICOS+ T cells correlated with the hemagglutination inhibition (HAI) titers induced upon influenza vaccination. Simian immunodeficiency virus (SIV) vaccine candidates administered to rhesus macaques induced long-lasting vaccine-specific Tfh cells which could be found for more than 53 weeks after the last vaccination dose; in vaccinated macaques, vaccine specific memory B cells correlated with the frequency of Tfh cells^19^. A vaccination trial with a recombinant Ebola vaccine candidate (rVSV-ZEBOV) showed that the frequency of Tfh cells increased after vaccination and that Th17-like Tfh cells increased more as compared to Th1- or Th2-like Tfh cells^7^.

Recent finding showed that the plasma levels of CXCL13, the chemokine responsible for migration of Tfh cells to the GCs, correlated with the amplitude of specific ab responses induced upon vaccination^20^. Thus CXCL13 in plasma could represent a potential biomarker to monitor vaccine responses in lymphoid tissue of humans which are otherwise inaccessible for large clinical studies. Higher CXCL13 plasma levels were also reported in various pathological conditions in humans^21–26^.

In the current study, we measured the ab responses to HBV vaccine provided with three vaccine doses under accelerated schedule of 4 weeks apart in HIV-1 infected children and healthy controls at 1 and 6 months post vaccination. We measured the frequency of cTfh cells and assessed the expression of IFN-γ, IL-2, IL-4 and IL-21 cytokines in these cells following stimulation of peripheral blood mononuclear cells (PBMCs) with hepatitis B surface antigen (HBsAg). We also measured the levels of CXCL13 in plasma to assess its potential as biomarker for GC activities and response to HBV vaccination.

## Results

### Anti-HBs abs in plasma decline at 6 months from the last vaccination in both HIV-1 infected children and healthy controls

**Figure 1:**
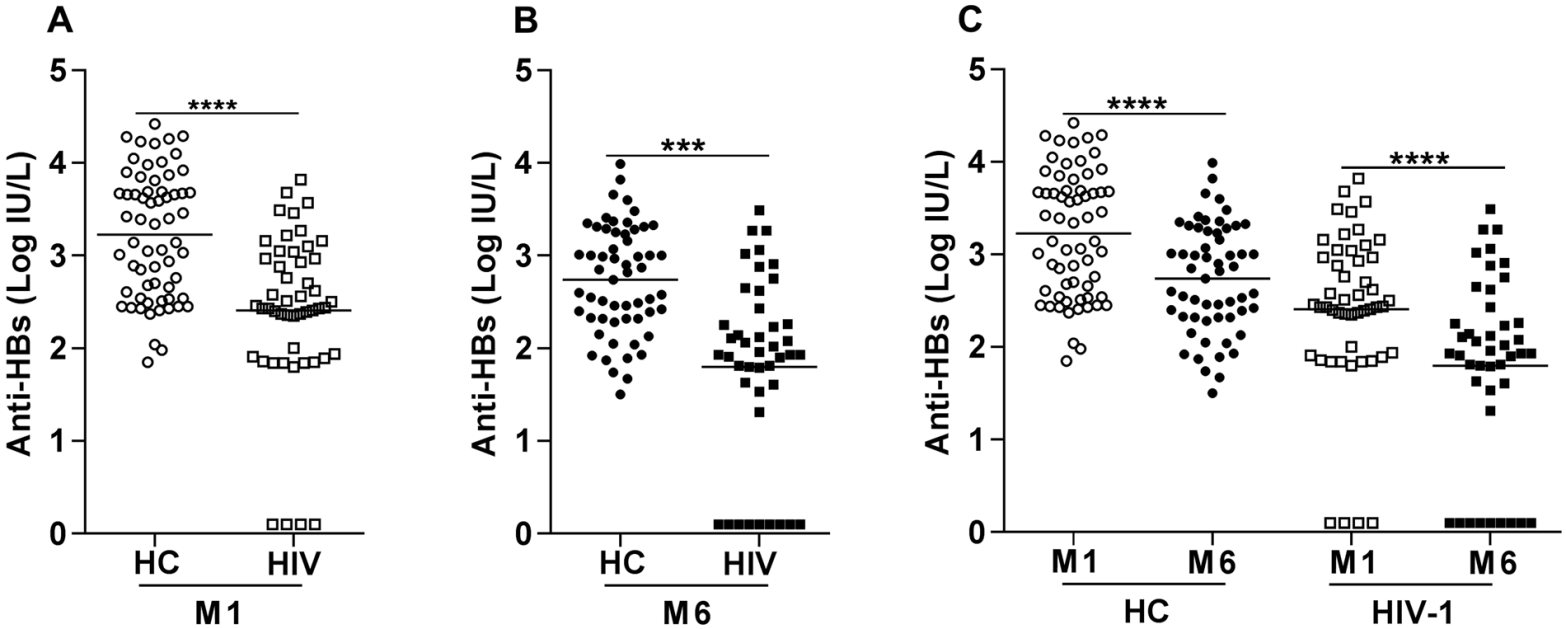
Anti-HBs ab levels in healthy controls and HIV-1 infected children at 1 month and 6 months post-vaccination. Anti-HBs abs were measured in plasma specimens from healthy controls (n = 63) and HIV-1 infected (n = 49) children at baseline and at 1 month (**A**) and 6 months (**B**) [healthy controls (n = 57) and HIV-1 infected children (n = 43) children] after vaccination. All children had undetectable anti-HBV ab levels before vaccination and after three doses of HBV vaccine, all children, except 4 HIV-1 infected (non-responder), responded with production of specific HBV abs. When ab titers to HBV vaccine were compared within the same group of healthy or HIV-1 infected children (**C**), a decline in ab titer was detected at 6 months from the last vaccination dose in both groups. Unpaired T test was used for panels A and B and paired T test for panel C. The lines represent the median values in the figures. ***p < 0.001 and ****p < 0.0001.

Plasma levels of anti-HBs abs were measured in specimens from HIV-1 infected children and healthy controls prior to and following vaccination. The plasma concentration of anti-HBs abs before vaccination was below the detection limit of the assay for all children included in the study. After one month from the last vaccination, 61 (96.8%) healthy controls and 35 (71.4%) HIV-1 infected children were high responders with titers of anti-Hbs abs between >2–4.4 log IU/L [Fig. 1A]. Among the vaccinated, 10 HIV-1 infected children (20.4%) and 2 healthy controls (3.2%) were weak responders with median titers between 1–2 log IU/L. Four HIV-1 infected children were non-responders with titers below 1 log IU/L.

Anti-HBs ab levels were significantly lower in HIV-1 infected children compared to healthy controls at 1 month from the last vaccination [median 2.44 (range: 0.1–3.8) log IU/L vs 3.3 (range: 1.9–4.4) log IU/L, P < 0.0001] [Fig. 1A]. We also compared the median levels of anti-HBs abs after 6 months from the last vaccination [Fig. 1B] in healthy controls (2.8 log IU/L, range: 1.5–4 log IU/L) and HIV-1 infected children (median 1.9 log IU/L, range: 0.1–3.5 log IU/L); the difference between the groups at this time point was also highly significant (P < 0.001). Nine (21%) of the HIV-1 infected children were found to be non-responders at 6 months; this pattern was not detected in any of the healthy controls. The levels of anti-HBs abs decreased significantly between 1 and 6 months from the last vaccination in both HIV-1 infected children (P < 0.0001) and healthy controls (P < 0.0001) [Fig. 1C].

### Similar frequency of cTfh cells before and after vaccination in HIV-1 infected and control children

**Figure 2:**
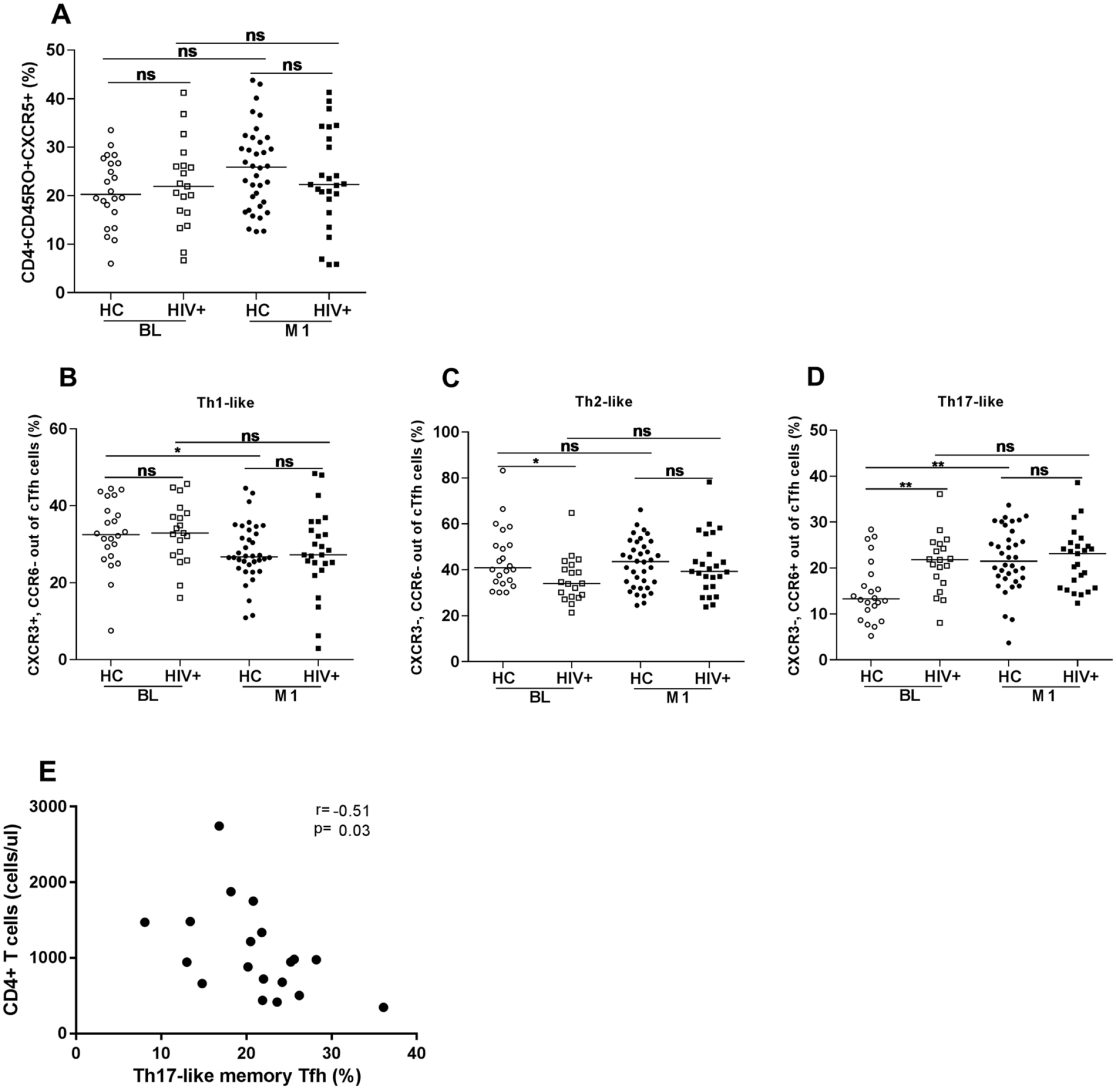
Frequency of cTfh cells and of Th1-, Th2- and Th17-like cTfh cells. The frequency of cTfh cells (**A**) and Th1-, Th2- and Th17-like cTfh cells (**B**–**D**) was measured in specimens from healthy controls and HIV-1 infected children. 22 healthy controls (HC) and 19 HIV-1 infected children were examined at baseline (BL) and 36 HC and 25 HIV-1 infected children after 1 month from last vaccination. A correlation between CD4+ T cell count and Th17-like cTfh cells was found at baseline in HIV-1 infected children (**E**). Unpaired and paired T test was used to calculate the differences between and within the groups. ns = not statistical significant, *p < 0.05, and **p < 0.01.

The gating strategy for identification of cTfh cells is illustrated in Supplementary Figure 1. We measured the frequency of cTfh cells before and after vaccination in HIV-1 infected children and controls and no significant difference was detected in the frequency of Tfh cells between the groups. The median frequency of cTfh cells prior to vaccination was 20.3% (range: 6.0–33.5%) for healthy controls and 20.8% (range: 6.7–41.2%) for HIV-1 infected children (Fig. 2A). At 1 month after vaccination, the median frequency of cTfh cells was 26.0% (range: 12.6–43.8%) in healthy controls and 22.3% (range: 5.8–41.3%) in HIV-1 infected children (Fig. 2A).

We also assessed the frequency of Th1, Th2 and Th17-like cTfh cells by characterizing CCR6 and CXCR3 expression on cTfh cells (Fig. 2B–D). The frequency of Th1-like cTfh (CXCR3 + CCR6−) was lower at 1 month post-vaccination as compared to baseline in healthy controls (31.8 vs 29.1%, P = 0.03). Prior to vaccination, a higher frequency of cTfh cells with a Th2-like phenotype (CXCR3 − CCR6−) was found in healthy controls compared to HIV-1 infected children (41 vs 34%, P = 0.01); no difference, was however, detected for this subset of cTfh cells at 1 month post-vaccination.

The frequency of Th17-like cTfh cells (CXCR3 − CCR6+) was significantly higher in HIV-1 infected children compared to healthy controls at baseline (median: 21.8 vs 13.3%, P = 0.007) [Fig. 2D]. After vaccination however, the frequency of Th17-like cTfh cells significantly increased in healthy controls compared to baseline (median: 13.3 vs 20.1%, P = 0.007), whereas the frequency of Th17-like cTfh cells remained similar when comparing baseline and 1 month post-vaccination in HIV-1 infected children (19.0 vs 21.8%, P > 0.05).

We then analysed the levels of anti-HBs abs in relation to the frequency of cTfh cells, CD4+ T cell count and length of ART treatment. A significant negative correlation was found between the CD4+ T cell count and the frequency of Th17-like cTfh cells in HIV-1 infected children prior to vaccination (r = −0.51, p = 0.03) (Fig. 2E).

### Cytokine expression in Tfh cells stimulated with HBsAg

PBMCs were cultured for 5 days in presence of the CD28/CD49d costimulatory complex alone or together with the HBsAg and the frequency of cTfh cells expressing IFN-γ, IL-2, IL-4 and IL-21 was measured. The gating strategy for the identification of cytokine expressing cTfh cells in HBsAg activated PBMCs is shown in Supplementary Figure 1.

**Figure 3:**
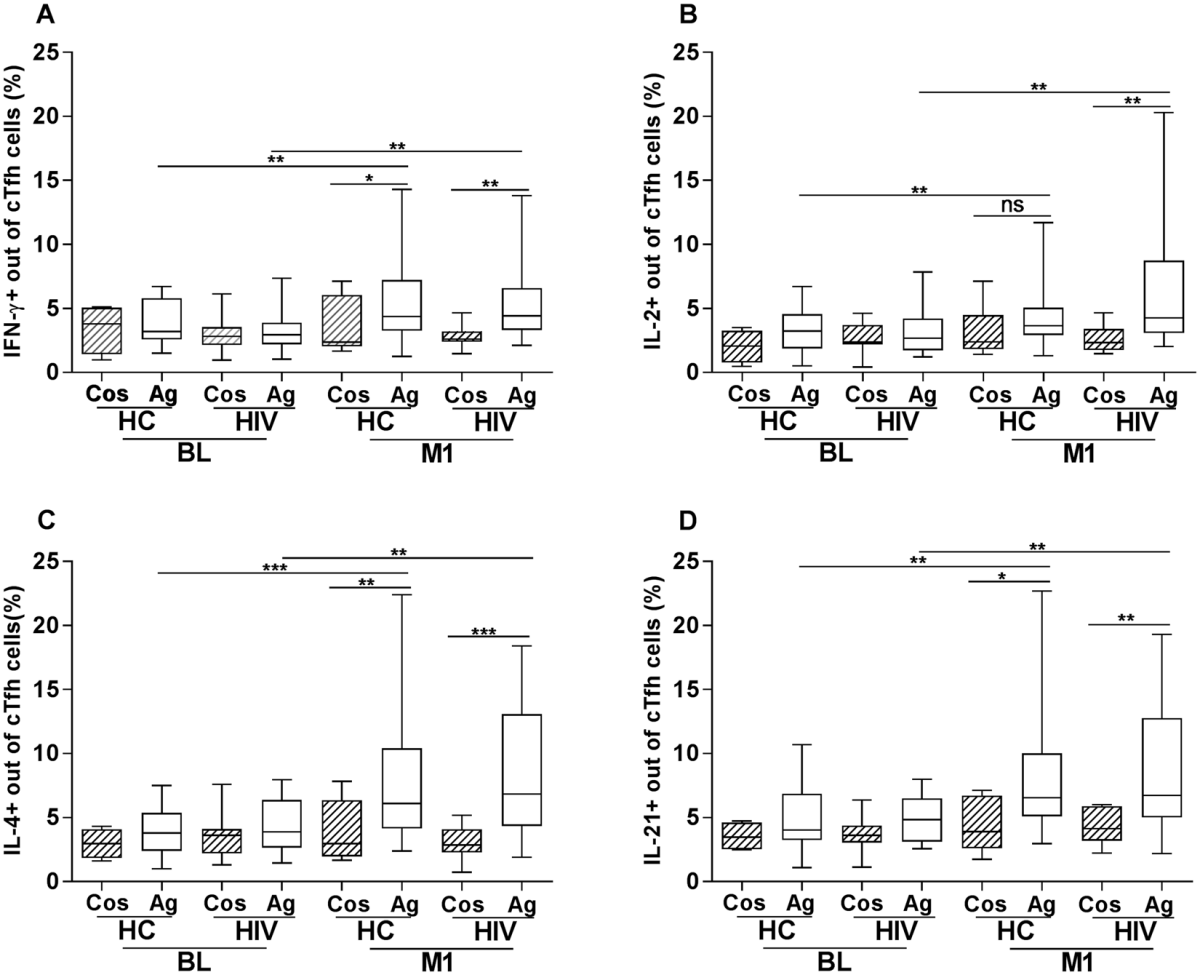
Expression of IFN-γ, IL-2, IL-4 and IL-21 by cTfh cells. The expression of cytokines IFN-γ, IL-2, IL-4 and IL-21 by cTfh cells in specimens from HIV-1 infected children and healthy controls after 5 days of culture is shown. Anti-human CD28/CD49d alone (Cos) or anti-human CD28/CD49d together with HBsAg protein (Ag) were added to the cultures. The expression of IFN-γ, IL-2, IL-4 and IL-21 among cTfh cells (Figure **A**–**D**) in response to HBsAg is shown for 20 healthy controls and 20 HIV-1 infected children at baseline (BL) and for 31 HC and 26 HIV-1 infected children at 1 month (M1) after vaccination. Samples from HC (BL = 4 and M1 = 9) and HIV-1 infected children (BL = 8 and M1 = 10) were used in culture stimulated with the CD28/CD49d complex. ns = not statistical significant, *p < 0.05, **p < 0.01 and ***p < 0.001.

At baseline, there was no difference between the frequencies of cTfh cells expressing cytokines in samples stimulated with the co-stimulatory complex alone or in presence of the HBsAg. Comparing the cytokine expression in cTfh cells in specimens from healthy controls and HIV-1 infected children stimulated with HBsAg, we found a similar frequency of cTfh cells expressing IFN-γ, IL-2, IL-4 and IL-21 between the two groups at baseline; the median frequency of IFN-γ was 3.2 vs 3.0%; IL-2, 3.2 vs 2.7%; IL-4, 3.8 vs 3.9% and IL-21, 4.0 vs 4.8% in healthy controls and HIV-1 infected children, respectively [Fig. 3A–D]. Cytokine expression in cTfh cells stimulated with the HBsAg protein significantly increased in specimens from 1 month after vaccination compared to baseline in both healthy controls and HIV-1 infected children. In healthy controls, the frequency of cTfh cells expressing IFN-γ (3.3 vs 5.5%, P < 0.01), IL-2 (3.4 vs 4.0%, P < 0.01), IL-4 (3.9 vs 6.1%, P < 0.001) and IL-21 (4.1 vs 7.9%, P < 0.01) was significantly increased in specimens obtained at 1 month post vaccination. Similarly, in specimens from HIV-1 infected children, the expression of IFN-γ (3.0 vs 4.4%, P < 0.01), IL-2 (2.7 vs 4.3%, P < 0.01), IL-4 (3.9 vs 7.0%, P < 0.01) and IL-21(4.8 vs 6.6%, P < 0.01) was increased significantly at 1 month post-vaccination compared with baseline. The cytokine expression in cTfh was comparable between healthy controls and HIV-1 infected children following vaccination [Fig. 3A–D]. In presence of HBsAg, the expression of IFN-γ, IL-4 and IL-21 increased significantly compared to stimulation with costimulatory complex alone. On the other hand, at 1 month post-vaccination, IL-2 expression increased to a significant level only in PBMCs from HIV-1 infected children treated with HBsAg [Fig. 3B], but not in healthy controls.

We analyzed whether there was a correlation between the length of pre-ART period, length of ART treatment, CD4+ T cell counts and viral load with the frequency of cytokine expressing cTfh cells at 1 month from vaccination in HIV-1 infected children. We could identify a positive significant correlation between the length of ART treatment and the frequency of IL-4 + cTfh cells (r = 0.42; p = 0.03).

### High levels of CXCL13 in plasma from HIV-1 infected children

**Figure 4:**
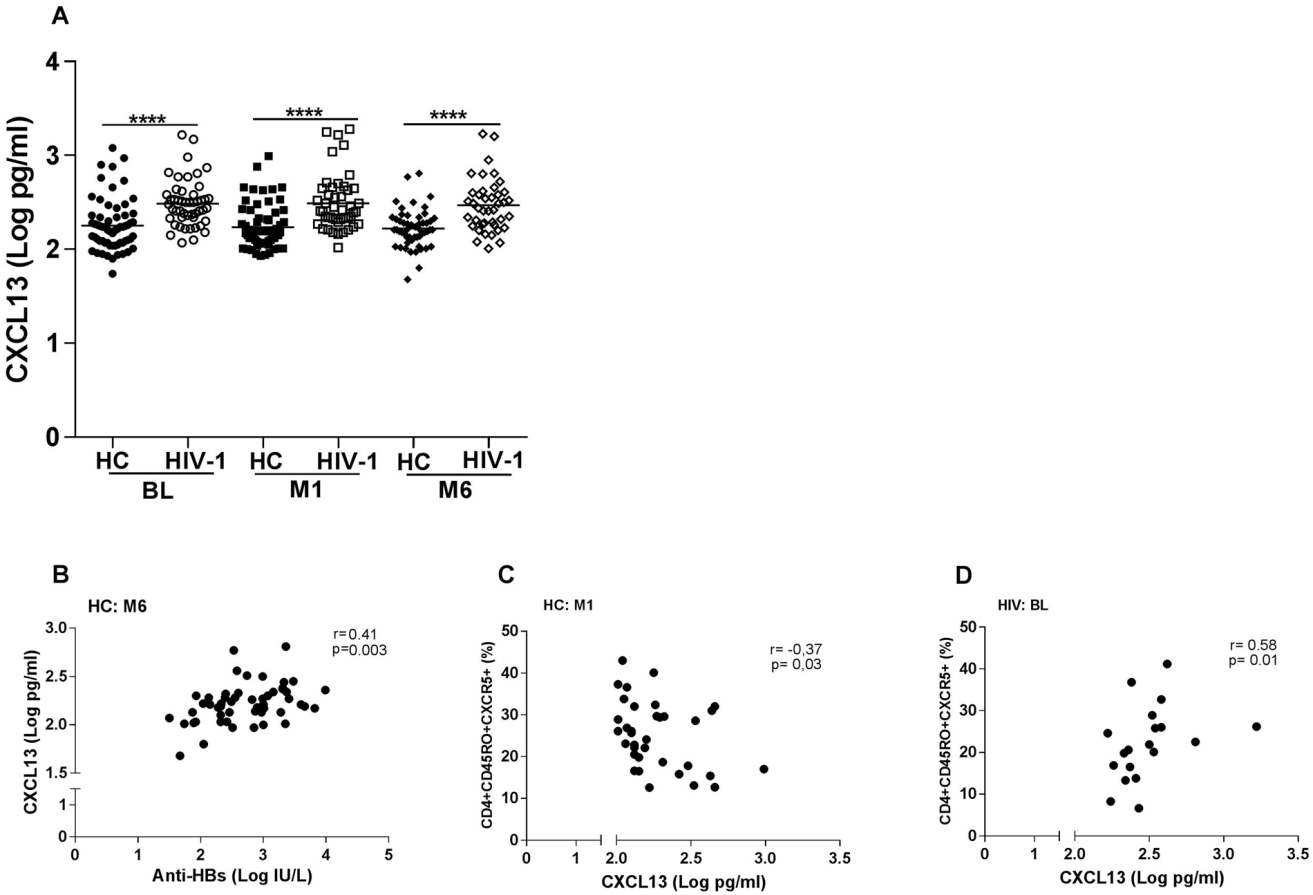
CXCL13 levels in plasma of healthy controls and HIV-1 infected children prior to vaccination and at 1 and 6 months post vaccination. CXCL13 was measured prior to vaccination and at 1 and 6 months post vaccination in specimens from healthy controls (n = 63, 62 and 53) and HIV-1 infected children (n = 49, 47 and 41) (**A**). ANOVA was used to calculate the differences between healthy controls and HIV-1 infected children at the 3 time points. Unpaired T test was also used to calculate the differences between healthy controls and HIV-1 at individual time points. The lines represent the median values in the figures. ****p < 0.0001. A correlation was found between the plasma levels of CXCL13 and anti-HBs abs at 6 months from the last vaccination in healthy controls (panel ﻿B﻿: n = 53). A significant correlation was found in the group of healthy controls at 1 month post vaccination (C; n = 35) ﻿and﻿ HIV-1 infected children when comparing the levels of plasmatic CXCL13 expression and frequency of cTfh cells at baseline (D; n = 19). HIV = HIV-1 infected children; HC = healthy controls.

Plasma levels of CXCL13 were measured before and after vaccination in HIV-1 infected children and healthy controls. The levels of CXCL13 were significantly higher (p < 0.0001) in HIV-1 infected children prior to vaccination (median: 2.5 log pg/ml; range: 2.1–3.2 log pg/ml) compared to healthy controls (median: 2.2 log pg/ml; range: 1.7–3.1 log pg/ml) [Fig. 4A]. There was no change in CXCL13 concentration after vaccination within the groups. HIV-1 infected children had a higher median of CXCL13 plasma level at 1 month and 6 months post-vaccination (p < 0.0001) compared to healthy controls (2.4 vs 2.2 log pg/ml and 2.4 vs 2.2 log pg/ml, respectively).

We correlated the levels of plasma CXCL13 with the frequency of cTfh cells, Th1-, Th2- and Th17-like Tfh cells and the levels of anti-HBV vaccine abs (Table 1). At 6 months from the last vaccination, plasma levels of CXCL13 were positively correlated with anti-HBs ab (r = 0.41, p = 0.003) [Fig. 4B] levels only in healthy controls. ﻿In healthy controls a negative correlation was found between the frequency of cTfh cells and the plasma levels of CXCL13 at 1 month post vaccination (r = −0.37, p = 0.03) [Fig. 4C].We found a positive correlation between the frequency of cTfh cells and plasma levels of CXCL13 in HIV-1 infected children prior to vaccination (r = 0.58, p = 0.01) [Fig. 4D] but this correlation was not found prior to vaccination in healthy controls.

**Table 1:** Correlation of plasma CXCL13 with sub-populations of Tfh cells and antibodies to HBV vaccine.

Parameters analyzed	CXCL13 HC	CXCL13 HIV-1
**At baseline**		
cTfh cells	ns	**r = 0.58**
		**p = 0.01**
Th1-like Tfh cells	ns	ns
Th2-like Tfh cells	ns	ns
Th17-like Tfh cells	ns	ns
**At month 1**		
cTfh cells	**r = −0.37**	ns
	**p = 0.03**	
Th1-like Tfh cells	ns	ns
Th2-like Tfh cells	ns	ns
Th17-like Tfh cells	ns	ns
Anti-HBs Abs	ns	ns
**At month 6**		
Anti-HBs Abs	**r = 0.41**	ns
	**p = 0.003**	

HC = healthy children; ns = not significant.

**Table 2:** Clinical characteristics of healthy controls and HIV-1 infected children included in the HBV vaccination study.

Characteristic	Controls (N = 63)	HIV-1 (N = 49)
Age (years): Mean (Range)	6.7 (4.0–8.0)	7 (4.0–9.0)
Gender		
Male	42 (66.7%)	25 (51.0%)
Female	21 (33.3%)	24 (49.0%)
MUAC		
<13.5 cm	—	5 (10.2%)
13.5–14.5 cm	5 (7.9%)	10 (20.4%)
>14.5 cm	58 (92.1%)	34 (69.4%)
CD4+ T cell count (cells/μl): Mean (Range)	ND	933.3 (195–2744)
WHO stage		
I	NA	27 (55.1%)
II		15 (30.6%)
III		4 (8.2%)
IV		3 (6.1%)
ART regimen		
First line		
AZT + 3TC + NVP	NA	39 (79.6%)
AZT + 3TC + EFV		3 (6.1%)
ABC + 3TC + EFV		4 (8.2%)
ABC + 3TC + NVP		1 (2.0%)
Second line		
ABC + ddl + LPV/r		1 (2.0%)
AZT + 3TC + LPV/r		1 (2.0%)
Months on ART: Mean (Range)	NA	41,8 (0.03–85)
Months pre-ART: Mean (Range)	NA	41.5 (2–100)
Viral load (Copies/ml)		
<150 copies/ml	NA	36 (73.5%)
150–999 copies/ml		4 (8.1%)
1000–57001 copies/ml		9 (18.4%)

ND = not done.

NA = not applicable. MUAC = Mid-Upper Arm Circumference.

## Discussion

The introduction of HBV vaccine in countries with a high burden of HBV infection has significantly reduced the prevalence of this infection and related complications^27^. Several clinical trials have previously shown that an accelerated vaccination schedule increases the likelihood of inducing high ab response in highly HBV endemic areas and in individuals who are at risk of acquiring HBV infection, especially immuno-compromised patients^28–33^. In our prospective vaccination study, healthy controls and HIV-1 infected children received three doses of HBV vaccine and 96% of the children responded to this vaccine with an increased titer of anti-HBV vaccine abs at 1 month from the last vaccination dose. Anti-HBs ab titers, however, declined significantly in both healthy controls and HIV-1 infected children at 6 months from the last vaccination and, at this time point, a significant proportion (21%) of HIV-1 infected children were non-responders with anti-HBs Abs titers lower than the cut-off value for protection. The rapid decline in protective abs to HBV vaccine in HIV-1 infected individuals is a known phenomenon which requests special attention; in this group of patients a second series of vaccination or increasing the vaccine dose may be alternative strategies^34, 35^. Despite the rapid decline in protection previously noticed following HBV vaccination and the recommendation formulated by experts to introduce a fourth booster of HBV vaccine in order to slow down the rapidly declining HBV vaccine ab levels^30, 36^, most African countries still provide only three doses of HBV vaccine.

T-cell dependent vaccine responses rely on the formation of specialized structures within the GC and the differentiation of ab producing cells^9^. In order to understand whether the cellular components involved in the GC may be affected in their phenotype and function during HIV-1 infection, we determined the frequency of cTfh cells in healthy controls and HIV-1 infected children. To our surprise, the frequency of cTfh cells was comparable in the two groups of HIV-1 infected children and healthy controls and was not affected by vaccination. We could not find a correlation between ab responses to HBV in HIV-1 infected and control children and frequency of cTfh cells. A correlation between cTfh cells and Ab responses has been reported for other T-cell dependent types of vaccines including influenza virus^11, 16^. Accordingly, it is possible that the engagement of cTfh cells and their activation upon vaccination may differ between vaccines. Our previous results revealed a lower frequency of cTfh cells in blood of HIV-1 infected children compared to healthy controls^37^. This discrepancy in the frequency of Tfh cells between the previously published and present studies could be due to an improved response to ART in HIV-1 infected children in Ethiopia, possibly by earlier ART initiation and increased adherence. Another study assessing influenza vaccine responses recently reported that the frequency of peripheral Tfh in HIV-1 infected and healthy children was similar between the groups and independent from the hemagglutination inhibition titers which were used to group HIV-1 infected children in responders and not responders^38^.

We also measured the frequency of Th1, Th2 and Th17-like cTfh cells using CXCR3 and CCR6 expression^13^. We found that the frequency of Th17-like cTfh cells was significantly higher in HIV-1 infected children compared to controls prior to vaccination and that the CD4+ T cell count and the frequency of Th17-like cTfh cells were negatively correlated. In the group of healthy controls included in our study, the frequency of Th17-like cTfh cells increased after vaccination. A similar observation was reported by Farooq *et al*.^7^, showing that rVSV-ZEBOV Ebola vaccine induces higher frequencies of Th17-like Tfh cells but not of Th1 or Th2 Tfh cells in humans. These findings should promote further investigations to assess the role that Th17-like Tfh cells may play in vaccine response.

We also addressed the capacity of cTfh cells to express cytokines *in vitro* in response to HBV antigenic stimulation and showed, for the first time, that cTfh cells expressed IFN-γ, IL-2, IL-4 and IL-21 upon stimulation with HBsAg. Litjens and collaborators^39^ studied how IFN-γ + CD4+ T cells and different subsets of memory CD4+ T cells obtained from HBV vaccinated individuals responded to stimulation with HBsAg. They showed that HBsAg specific IFN-γ producing CD4+ T cells were significantly higher in vaccinated compared to non-vaccinated healthy adults. In our study, the frequency of cTfh cells expressing cytokines in response to HBsAg significantly increased in both HIV-1 infected children and controls after vaccination. Several studies confirmed the role of IL-21, produced by Tfh cells, to induce B cells differentiation into memory B cells and ab-producing cells^40, 41^. In IL-21 knockout mice, the formation of GC is normal but the GC reaction declines rapidly and affects ab production^42, 43^. Studies performed in IL-4 knockout mice showed that lack of IL-4 resulted in reduced IgG1 production due to loss of class switching function and that blocking the IL-4Rα reduced plasmablast density in the lymph nodes^44^. Although IL-4 and IL-21 cooperate for efficient ab production and GC reaction^45^, these two main Tfh cell cytokines work independently from one another as IL-21 is expressed earlier than IL-4; efficient GC B cells somatic hypermutation is mediated by IL-21 and plasma cell differentiation and class switching by IL-4^46^. The production of IL-21, however, influences IL-4 levels as shown by reduced IL-4 production in IL-21 knockout mice. In the present study, the decline in anti-HBs abs’ levels following vaccination was more pronounced in HIV-1 infected children; however cTfh cells from HIV-1 infected and control children once exposed to HBsAg showed similar IL-21 and IL-4 expression. These results suggest that the function of other cells important to sustain ab response may be impaired in HIV-1 infection. The IL-4 expression in HBsAg stimulated cTfh cells from vaccinated HIV-1 infected children showed a correlation with the length of ART treatment illustrating the beneficial effect of ART on parameters of vaccine responses.

CXCL13, mainly secreted by follicular DCs, is a chemoattractant critical for the distribution of lymphocytes within the GC^20^. In this study, elevated plasma CXCL13 concentration was measured in HIV-1 infected children compared to controls at baseline. It has been previously reported that elevated plasma CXCL13 levels can be found during HIV-1 infection produced by activated monocytes and that increased CXCL13 production during HIV-1 infection may lead to impairment of B cell function^20, 47^. In the present study we could not detected any change in CXCL13 concentration after vaccination in either control or HIV-1 infected children. Havenar-Daughton and collaborators^20^ reported an increase in plasma CXCL13 concentration in two cohorts of non-HIV infected individuals following vaccination with either yellow fever virus vaccine or with a candidate HIV vaccine. A positive correlation was also reported between the frequency of GC Tfh cells and plasma CXCL13 concentration in the latter study^20^ in a small number of HIV-1 infected and non-infected individuals. It is interesting that, prior to vaccination, there was a positive correlation between CXCL13 and the frequency of cTfh cells in the group of HIV-1 infected subjects in our study which could not be demonstrated in the group of healthy children. We also found a positive correlation between CXCL13 and the titers of anti-HBs abs in healthy controls at 6 months post-vaccination. Further studies are needed to confirm the validity of monitoring GC functions during vaccination in humans by measuring CXCL13 in plasma and to clarify whether the high CXCL13 level in the serum of HIV-1 infected patients promotes or disrupts the capacity of the individuals to respond to vaccination.

In conclusion, our results showed that three doses of HBV vaccine induced protective titers of anti-HBs abs at 1 month after vaccination in HIV-1 infected children which rapidly declined at 6 months post-vaccination, suggesting the need for an additional vaccine dose to booster the titers of specific abs in circulation. The capacity of cTfh cells to respond to stimulation with HBV antigens is comparable between HIV-1 infected and control children. Further studies are accordingly needed to pin-point the molecular and immunological mechanisms responsible for reduced Ab production in HIV-1 infected children receiving HBV vaccine.

## Method and Materials

### Study design and participants

The study was conducted at Zewditu Memorial Hospital and the Pediatric department of the All Africa Leprosy, Tuberculosis and Rehabilitation Training Center (ALERT) in Addis Ababa, Ethiopia. Age matched healthy controls were recruited from two child care centers in Addis Ababa (Muday Charity organization and SOS village). A total of 158 children, 93 HIV-1 negative and 65 HIV-1 positive, were enrolled in the study. All participants underwent an initial screening for previous or active HBV infection; children showing previous or active infection with HBV were excluded from the study. HBV vaccine was administrated intramuscularly to a total of 63 non HIV-1 infected controls and 49 HIV-1 infected children; all HIV-1 infected children received antiretroviral treatment (ART). Clinical parameters were collected from all children (Table 2).

The project proposal was reviewed and approved by the ethical review committees of the Armauer Hansen Research Institute (AHRI) Addis Ababa, the IRB of College of Health Sciences, Addis Ababa University, Addis Ababa Health Bureau Ethics Board and the National Research Ethics Review Committee of Ethiopia. Written informed consent was obtained from the parents or guardians of study participants following a clear explanation of the study purpose, benefit and possible discomfort. The ethical committee at the Karolinska Institutet approved the study of the patient material collected during the vaccination study in Addis Ababa. All experiments were performed in accordance with relevant guidelines and regulations.

### Vaccine administration and blood sampling

The vaccine used in our study was the Hepatitis B vaccine (rDNA), (Serum Institute of India, India) which consists of purified HBsAg. All children received 3 doses (each of 10 μg) with 4 weeks interval (accelerated vaccination schedule) in between the doses^30^. There was no severe adverse event reported upon vaccination in either healthy controls or HIV-1 infected children. Blood (3–5 ml) was collected at baseline (prior to vaccination) and at 1 and 6 months from the last dose of vaccine. Six children in the group of HIV-1 infected and 6 healthy controls were lost for sample collection at 6 months.

PBMCs were isolated by Ficoll gradient centrifugation and stored in 90% fetal bovine serum (Sigma-Aldrich, USA) and 10% dimethyl sulfoxide (DMSO) (Sigma-Aldrich, USA) in liquid nitrogen at −196 °C. Plasma specimens were frozen at −80 °C until further analyses were conducted.

### Determination of plasma anti-HBs abs and CXCL13 titers by ELISA

The HBsAg EIA 3.0 enzyme immunoassay and the anti-hepatitis B nucleocapsid core (anti-HBc) antigen monolisa assay (Bio-Rad, France) were used to exclude active or previous HBV infection. The anti-HBs Monolisa Plus assay (Bio-Rad, France) and Human CXCL13 DuoSet ELISA (R&D systems, Minneapolis, MN) were used to measure the plasma levels of anti-HBs abs and CXCL13 chemokine, respectively. The assays were run according to the instruction provided by the manufacturers and all samples and standards were tested in duplicate. The OD values were converted to concentrations using Microplate manager version 6 (Bio-Rad, California, USA).

### Plasma HIV-1 viral load determination

RNA was extracted from plasma using an automated m2000sp Abbott Real-Time HIV-1 assay system following the manufacturer’s protocol (Abbot Laboratories, Abbot Park, IL). Plasma HIV-1 RNA levels were measured using Quantitative Real-Time HIV-1 assay by the Abbott m2000rt instrument with the lower detection limit of 40 copies/ml.

In the present study, 73.5% (36) of the HIV-1 infected children had levels of plasma HIV-1 RNA below 150 copies/ml prior to vaccination and only 18.4% (9) of them had over 1000 copies/ml. At 1 month post-vaccination all children with HIV-1 RNA below 150 copies/ml at baseline displayed HIV-1 RNA below 150 copies/ml, except 4 children with over 1000 copies/ml after vaccination.

### Characterization of cTfh cells

PBMCs were thawed and washed in complete RPMI-1640 medium supplemented with L-glutamine, in the presence of 10% FCS and penicillin-streptomycin (Thermo Scientific, South Logan, Utah, USA). To characterize cTfh cells, defined as CD4 + CD45RO + CXCR5+ T cells, PBMCs were stained with the following monoclonals: V500 anti-CD4 (L200), FITC anti-CD45RO (UCHL1), PerCp/Cy5.5 anti-CXCR5 (RF8B2), APC anti-CXCR3 (1C6/CXCR3), PE-Cy7 anti-CCR6 (11A9), all from BD Biosciences, and LIVE/DEAD® Fixable Near Infrared dead Cell Stain (Molecular Probes by Life Technologies, OR, USA). To assess the cytokine expression of cTfh cells in response to stimulation with HBsAg, PBMCs were cultured at a concentration of 1.5 × 10^6^ cells/ml with either the BD FastImmune CD28/CD49d (BD, San José, CA, USA) costimulatory complex or with CD28/CD49d complex together with the HBsAg (Subtype adw9; Fitzgerald, Concord, MA, USA)^39^. Cells were cultured for 5 days and 1 μl/ml Golgistop (BD, San José, CA, USA) was added to all culture conditions during the last 6 hours of incubation to block release of cytokines from cells. Thereafter cells were first stained for cell surface markers with the following monoclonals: Texas red anti-CD4 (RFT-4g; Abcam, Cambridge, UK), FITC anti-CD45RO (UCHL1; BD), PE-Cy7 anti-CXCR5 (J252D4; BioLegend, San Diego, CA, USA) and LIVE/DEAD® Fixable Near-IR dead Cell Stain (Life Technologies, Eugene, OR, USA). Cells were fixed and permeabilized using the BD Cytofix/Cytoperm kit according to the protocol from the manufacturer and thereafter stained with BV421 anti-IL-2 (5344.111), APC anti-IL-4 (MP4-25D2), BV711 anti-IFN-γ (B27) all from BD and PE anti-IL-21 (3A3-N2) from eBioscience (San Diego, CA, USA).

Cells were fixed in 2% paraformaldehyde; the samples were acquired in the LSR-II flow cytometer (BD Biosciences) and analyzed using FlowJo V9.8 (Tree Star Inc., Ashland, Oregon, USA).

### Data analysis and interpretation

Statistical analysis of the clinical characteristics was computed using a SPSS version 23 (IBM Corp, Armonk, NY, USA). The GraphPad prism version 7 (La Jolla, CA, USA) was used for ELISA and flow cytometry data analyses. ANOVA and t-test were used to assess the differences between HIV-1 infected and healthy controls pre and post-vaccination. Spearman correlation was also used to demonstrate the relationship between two continuous variables. A P value less than 0.05 was considered statistically significant.

### Data availability statement

We will make upon request materials, data and associated protocols promptly available to readers.

## Electronic supplementary material

Supplementary Figure 1
