# Supplementary figures and images for: T follicular helper cells and antibody response to Hepatitis B virus vaccine in HIV-1 infected children receiving ART
Source: Sci Rep. 2017 Aug 21;7:8956. doi: 10.1038/s41598-017-09165-6 (PMC5566956; doi:10.1038/s41598-017-09165-6)

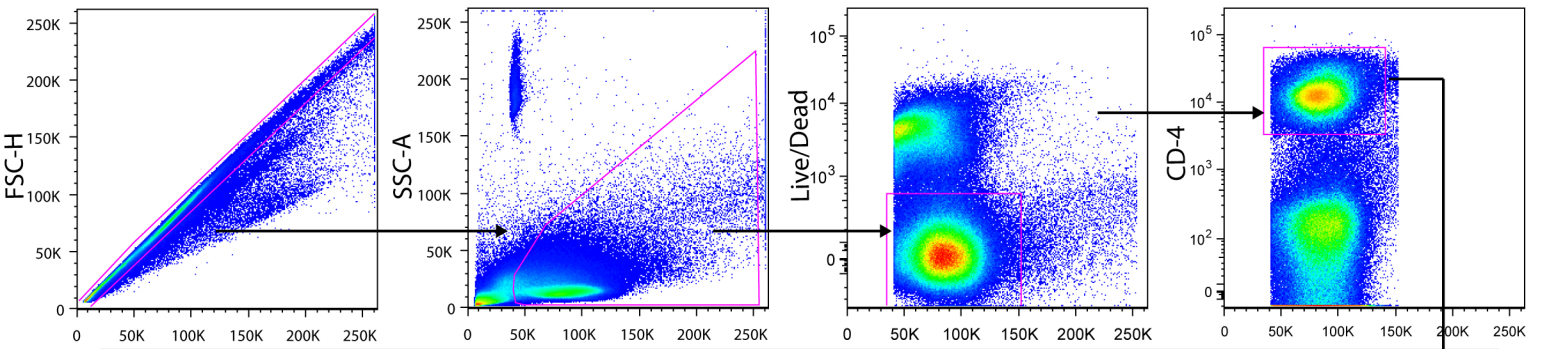

FSC-A

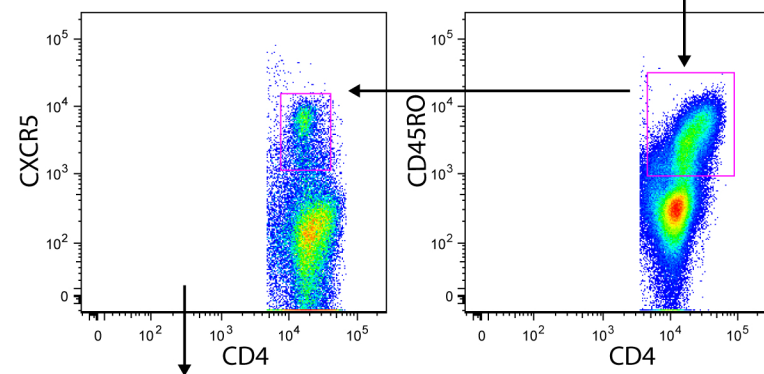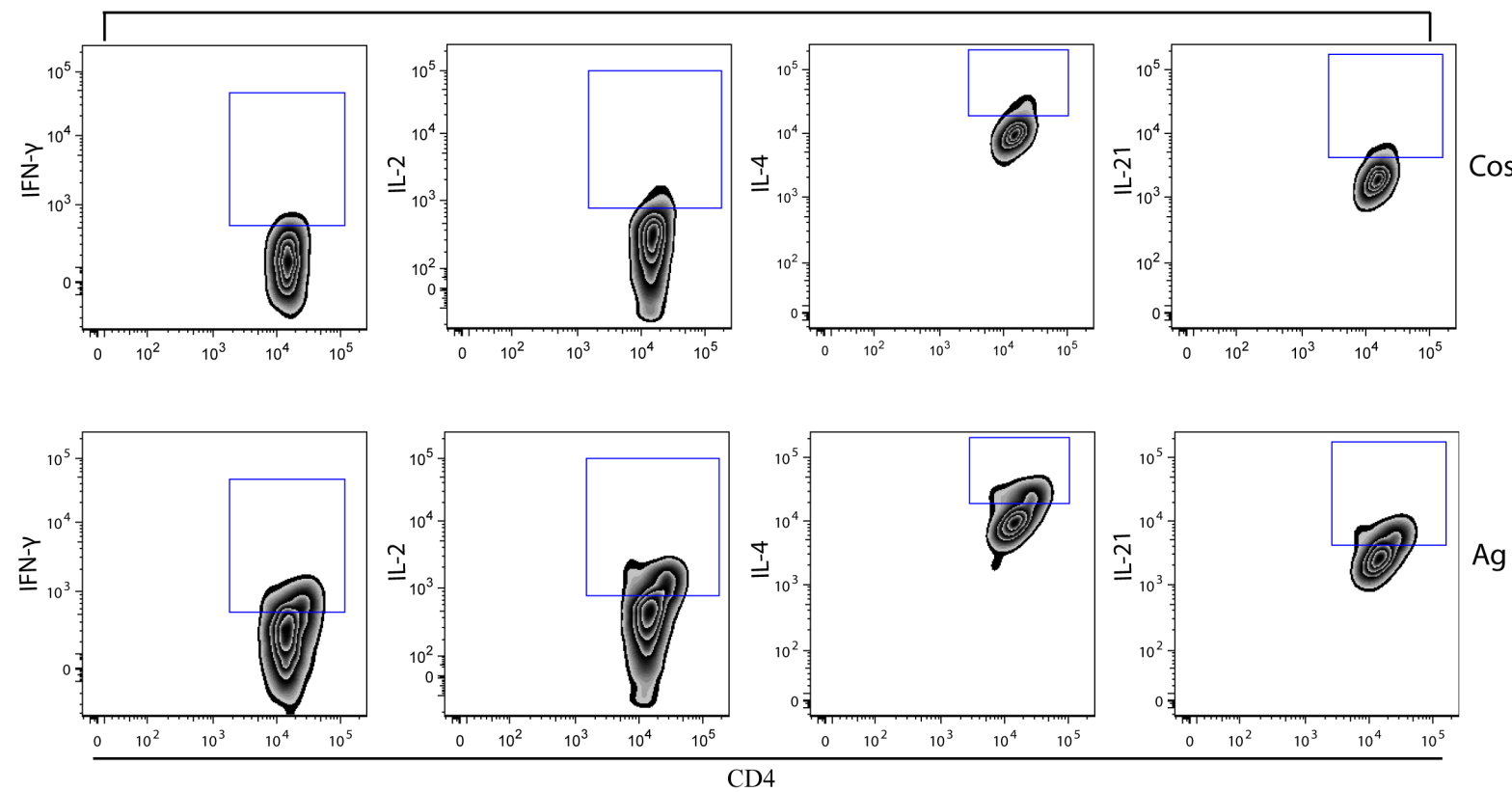

Supplement: Supplementary file 1 — Supplementary Figure 1 [file 41598_2017_9165_MOESM1_ESM.pdf]
